# Modeling of charged-particle multiplicity and transverse-momentum distributions in *pp* collisions using a DNN

**DOI:** 10.1038/s41598-022-11618-6

**Published:** 2022-05-19

**Authors:** E. Shokr, A. De Roeck, M. A. Mahmoud

**Affiliations:** 1 Physics Department, Faculty of Science, Mansoura University, Mansoura, Egypt; 2 CERN, Geneva, Switzerland; 3 Center for High Energy Physics (CHEP-FU), Faculty of Science, Fayoum University, El Faiyûm, Egypt; 4 Academy of Scientific Research and Technology (ASRT), Cairo, Egypt

**Keywords:** Physics, Particle physics, Experimental particle physics

## Abstract

A machine learning technique is used to fit multiplicity distributions in high energy proton-proton collisions and applied to make predictions for collisions at higher energies. The method is tested with Monte Carlo event generators. Charged-particle multiplicity and transverse-momentum distributions within different pseudorapidity intervals in proton-proton collisions were simulated using the PYTHIA event generator for center of mass energies $\sqrt{s}$= 0.9, 2.36, 2.76, 5, 7, 8, 13 TeV for model training and validation and at 10, 20, 27, 50, 100 and 150 TeV for model predictions. Comparisons are made in order to ensure the model reproduces the relation between input variables and output distributions for the charged particle multiplicity and transverse-momentum. The multiplicity and transverse-momentum distributions are described and predicted very well, not only in the case of the trained but also in the case of untrained energy values. The study proposes a way to predict multiplicity distributions at a new energy by extrapolating the information inherent in the lower energy data. Using real data instead of Monte Carlo, as measured at the LHC, the technique has the potential to project the multiplicity distributions for different intervals at very high collision energies, e.g. 27 TeV or 100 TeV for the upgraded HE-LHC and FCC-hh respectively, using only data collected at the LHC, i.e. at center of mass energies from 0.9 up to 13 TeV.

## Introduction

Inclusive particle multiplicity distributions are among the most basic global characteristics of high energy proton-proton (*pp*) collisions^1^, but have been proven to be difficult to describe or predict by standard Monte Carlo generator programs, such as PYTHIA^2^ and HERWIG^3^. The *pp* charged-particle multiplicity has been studied theoretically and experimentally at the Large Hadron Collider (LHC) in different experiments and for various colliding center of mass (CM) energies ($\sqrt{s}$)^1,4–10^. Charged-particle multiplicity distributions generated in these collisions in restricted pseudorapidity intervals ($|\Delta \eta |$), i.e. the probability $P(N_{ch},\sqrt{s},|\Delta \eta |)$ for producing the number of charged-particles in the final state ($N_{ch}$), depends on the number of interactions between quarks and gluons confined inside the colliding protons, and the underlying mechanisms of particle production^11^.

At LHC energies, *pp* interactions are dominated by soft QCD processes, i.e. interactions with small transverse-momentum ($$p_{T})$$ transfer. Such interactions cannot be treated perturbatively but are modeled phenomenologically^12^. These processes are very useful for studying QCD in non-perturbative regimes, tuning event generators and constraining the dynamics in phenomenological models. As the collision energy increases, the contributions from hard scattering processes increase which can be treated perturbatively. A generic term for such an experimentally collected event sample containing all produced events—soft and hard—is a minimum bias (MB) event sample. This is by itself is not a physics but an operational definition: the exact composition of the sample depends on the (minimum) requirements imposed to select the events in the experiment (e.g. it can be based on the amount of energy or number of particles observable in the experiment).

At the LHC, PYTHIA and HERWIG are the commonly used generators to describe the *pp* multiplicity distributions at the various center of mass energies at which the collider has operated over the past years. Comparisons to data at the different CM energies show that it is very challenging to describe the charged-particle multiplicity distributions with these models, despite the many tunable parameters available for the user. Moreover, we cannot be sure how well these parameters allow to cover the underlying dynamics and its energy dependence of in particular these soft processes. Sufficiently accurate descriptions of multiplicity distributions are however important at hadron colliders where we can have, now and in the future, about 20 to perhaps a few hundred of such minimum bias events per bunch crossing overlapping with a collision of interest. These additional events add significantly to the occupancy in the detectors and affect systematical uncertainties of precision measurements. As soon as such future hadron colliders turn into operation the characteristics of MB events will be measured in a very early stage of the operation, but until then, studies on the capabilities of such a new machine will have to rely on model predictions.

Therefore we present in this study an alternative approach where we make no prior assumption on any underlying Monte Carlo generator model or tuning of parameters, but use a machine learning technique to construct “the model”. This is similar to the very successful parton density distribution (PDF) determinations technique used by the NNPDF collaboration^13^, where instead of imposing explicit functional forms for the distributions at a starting scale, a neural network is used to provide that information, in order to reduce the source of potential bias from the initial assumptions.

The $p_T$ spectrum of final state charged hadrons is also an important observable in describing particle production in *pp* collisions^14^. As an example, the study of the $p_{T}$ spectrum in *pp* collisions offers a reference for the measurements of the suppression of high-$p_T$ particles (Jet Quenching) in a dense QCD medium produced in ion-ion collisions^15,16^. A solid knowledge of the rates and characteristics of the particle production are mandatory to distinguish e.g. rare soft processes from the relatively huge backgrounds of hadronic interactions^17^, which is one of the greatest challenges in these pursuits, and for extracting precision measurements from the data.

Since several years, particle physicists have continued to explore techniques to increase the analyzing power for measurements by using algorithms implementing multiple variables simultaneously. These so-called multivariate analyses techniques^18–20^ have been shown to provide significant support for different challenges in data analysis but also have some important limitations, with increasing dimensionality of the problem.

The implementation of these advanced analysis techniques, such as Machine Learning (ML), the increasing computer power and tailored processors for the problem, and the emergence of Deep Learning (DL) techniques around 2012^21^ provided tools that allowed to tackle complex problems without these previous limitations. In high energy physics, machine learning algorithms and techniques have been embraced early on for analyzing and collecting the huge amount of data produced by colliders^18^; e.g. the LHC is presently one of the largest data volume generators. The role of these new powerful techniques is clear, namely revolutionizing the handling and interpretation of these huge data volumes, and allowing to extract detailed physics results with increased sensitivity. These techniques are now considered essential tools at the LHC and have found important applications in data analyses, calibration, event triggering, flavor tagging, etc..^20,25–31^.

Recently, different algorithms and techniques based on Artificial Neural Networks, Genetic Programming and Machine Learning have been implemented for the studies as proposed in this paper, namely trying to explain, and modeling of, multiplicity distributions of hadron-nucleus^22^ and *pp* interactions^23,24^. The motivation to use Artificial Intelligence and Deep Neural Networks (DNN) for such studies is its ability to learn the complex relation between input interaction variables and output observables that arise in *pp* collisions since such interactions are hard to describe due to the absence of the information on how to describe the quantity of interest with the relevant interaction variables mathematically^19^, that the foundations for these techniques were proposed in^32–48^.

The test we propose is to check to what extend suitable DNNs will allow to predict e.g. the multiplicity distributions at other center of mass energies than those used in the learning process and provided no (significant) new physics processes set on in the new energy regime. In the example studied in this paper we use the multiplicity distributions of charged-particles simulated at energies where LHC collider has collected data. We check the ability to predict such distributions for both intermediate new energies and in a new regime reachable by possible future higher energies. Such higher energy extension could come from the CERN *pp* program by a High-Energy LHC Collider (HE-LHC) at e.g. 27 TeV that could be located in the present LHC tunnel, and be based on Future Circular Collider (FCC-hh) magnet technology currently under development^49^. Furthermore, we include the proposed 100 TeV FCC-hh^50^, potentially to be built using a new accelerator ring with 100 km circumference. The predictions are obtained using LHC simulations for 0.9, 2.36, 2.76, 5, 7, 8 and 13 TeV as input to the model training, i.e. CM energies at which the LHC has operated so far.

The strategy of this study is as follows. This study is a proof of principle of the underlying idea using the PYTHIA event generator instead of real data. This has the advantage that a uniform analysis can be performed with the “data sets” of all CM energies and that these are also available to be used as inputs. Charged-particle multiplicity distributions from LHC data are not available yet for all CM energies.

We set up a machine learning configuration and train the network with the *pp* multiplicity and transverse-momentum distributions of charged-particles generated using the PYTHIA event generator for seven increasingly wider pseudorapidity intervals and for different center of mass energies corresponding to the energies that the LHC operated at untill 2018. We use corresponding CM energy settings for data sets that may be collected in the future to test and support our proposed technique. We check the quality of the resulting model’s ability to predict generator distributions at different CM energies, including how well these interpolate between the measurements already made and how well they can predict distributions for higher energies.

As mentioned, a practical application for a real world prediction would require to use as input actual measurements based on data. At this point in time, these measurements have not been conducted for all CM energies at which the LHC was operated. Minimum bias charged-particle multiplicities distribution measurements do exist, and have been provided in particular by the CMS and ALICE collaborations over the last years. We hope that studies such as this one will strongly encourage that such measurements will be performed and published in future. Using such a method for predicting higher energies has the obvious drawback that if a strong new physics process will set on in between the region of the measurements and the new energy, this method will obviously not make a correct prediction. But turning this argument around: such deviations, when compared with the future data can then point to something new!

This paper has six further sections. Section “Deep Neural Network“ introduces the basics of the DNN. Section “Data preparation“ gives a summary of our method to collect and preparing data. Section “Prediction network“ explains in detail our model for predictions. Sections “Results and discussion“ and “Conclusion“ discuss the results and the conclusion respectively.

## Deep neural network

In ML modeling, an approximating function that describes the relation between inputs and outputs can be inferred automatically from the input data without providing explicit information about this function. The most powerful technique to infer an approximation *f*(*x*, *w*) of the unknown function *f*(*x*) is called supervised learning, in which the training process contains datasets that include inputs and the corresponding targets (desired outputs). The goal of learning is to determine the parameters *w* of the model, so we can obtain a functional approximation of the desired input-output map. In high energy physics, the training data is generally obtained from Monte Carlo simulations^18^.

**Figure 1:**
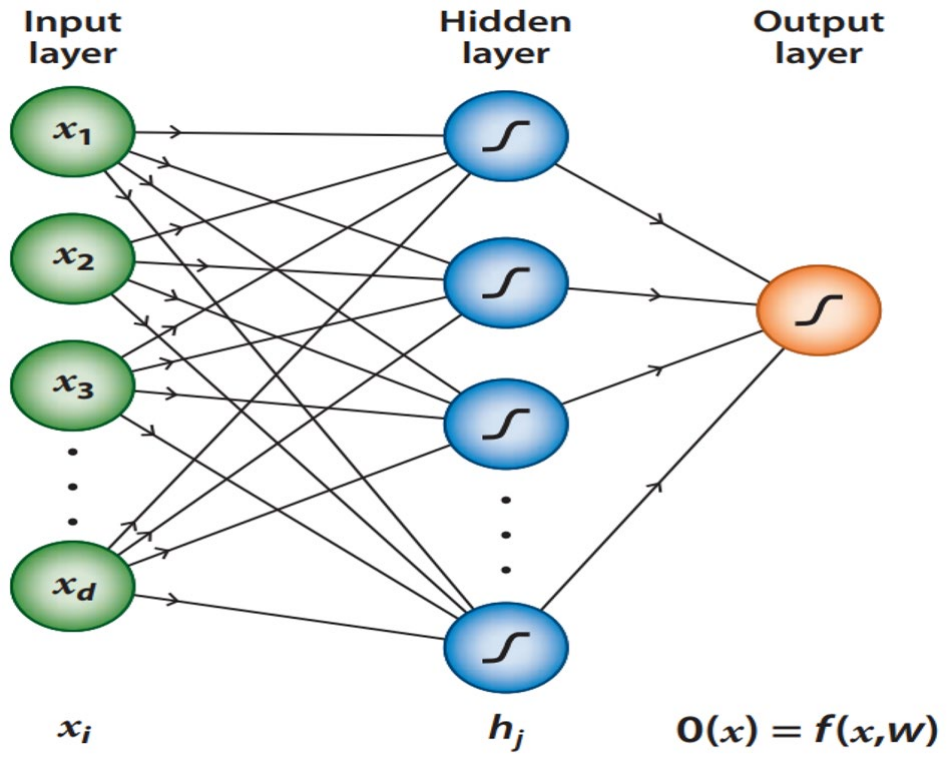
A simple feed-forward Neural Network with three layers, from^18^.

Feed-forward Neural Networks are the most popular and widely used multivariate methods^18^. It contains an interconnected group of neurons ordered in sequential layers, where each neuron has a role to process the received information with what is called an activation function, see section “Results and discussion”, then the result is moved to the next layer of nodes. The first layer, which receives the input variables is called the input layer, followed by one or more hidden layers. The last layer is responsible for the final response of the neural network and is called the output layer. Each interconnection is specified by a weight and a bias, which are the network parameters that are being learned and updated during the training process. A simple NN is shown in Fig. 1.

In Fig. 1, showing a NN that consists of one hidden layer of nodes and an input data layer with *d* feature variables (inputs) $x = \{x_{1} , x_{2} , . . . x_{d} \}$, the output of this network is

1 $$\begin{aligned} f(x,w)= g\left( \theta + \sum _{j}^{} w_{j}b_{j}\right) \end{aligned}$$

where *g* represents the activation function and $b_{j}$ is the output from the hidden neurons:

2 $$\begin{aligned} b_{j}= g\left( \theta _j + \sum _{i}^{} w_{ij}x_{i}\right) \end{aligned}$$

Where $\theta _j$ neuron bias, i is input number, and j is neuron number.

## Data preparation

PYTHIA^2^ is a general-purpose Monte Carlo event generator that is actively used in particle physics in general, and for the LHC in particular. This generator has undergone decades of development and tuning to collider and other data.

The event generation consists of several steps starting typically from a hard scattering process, followed by initial- and final-state parton showering, multi-parton interactions, and the final hadronization process. PYTHIA uses different model approaches for these steps, e.g. it uses a $p_{T}$-ordered perturbative approach^51^ for modeling of parton shower. The original impact parameter model^52^ for multi-parton scattering and the Lund string fragmentation model^53,54^ are used for the hadronization (fragmentation) of partons into hadrons.

The proton-proton collisions are generated in this work with the PYTHIA 8.186^55^ version of the program. The collisions are generated at $\sqrt{s} = 0.9, 2.36, 2.76$, 5, 7, 8, and 13 TeV, corresponding to the energies at which the LHC was operated from 2010 till 2018, in order to train and evaluate the model performance, and at the energies $\sqrt{s} = 10, 20, 27, 50, 100, 150$ TeV in order to compare with the prediction of our model and to show its ability to predict event distributions at the energies that were not used to train on. Different model response functions are extracted for different pseudorapidity intervals. In total 50*$10^6$ collisions were simulated at 7, 8 and 13 TeV, and 5*$10^6$ events were generated for other CM energy values, using default minimum bias generation settings of the generator, discussed below. The difference in the number of events was chosen to emulate the experimental situation where much larger data sets were collected at 7, 8 and 13 TeV at the LHC, than for the other CM energies.

The inelastic (diffractive and non-diffractive) proton-proton collisions were simulated using the PYTHIA Monash 2013 tune^56^. The Monash parameters are tuned such that these provide a reasonable description of the experimental data at LHC energies for the bulk of the minimum bias charged multiplicity distribution and several other event characteristics.

Minimum bias events and particles are selected in this study according to the following criteria. Each event must have at least one charged-particle in the final state which is emitted within the studied pseudorapidity interval and within the full acceptance of the azimuthal angle ($\phi$), and with a minimum $p_{T}$ > of 400 MeV. The number of events that pass those selection criteria at the different energies and pseudorapidity intervals $|\Delta \eta |$, i.e. starting the count of the number of particles from $\eta =$ 0 to the different $\eta$ limits at the negative and positive sides of the detector, are given in Table  1.

**Table 1:** The number of events that pass the selection criteria at different energies and different pseudorapidity interval limits; m stated for million.

$\sqrt{s}$	The number of events *$10^6$ at $|\Delta \eta |$						
	0.5	1	1.5	2	2.5	3	3.5
0.9	2.9221	3.6344	3.9446	4.1190	4.2369	4.3264	4.3994
2.36	3.1444	3.7824	4.0500	4.1987	4.2999	4.3785	4.4435
2.76	3.1794	3.8044	4.0659	4.2105	4.3092	4.3854	4.4490
5	3.3099	3.8861	4.1253	4.2576	4.3471	4.4163	4.4745
7 (5m)	3.3748	3.9275	4.1567	4.2822	4.3673	4.4334	4.4893
7 (50m)	34.572	39.890	41.942	43.038	43.792	44.399	44.928
8 (5m)	3.4016	3.9440	4.1688	4.2917	4.3753	4.4402	4.4951
8 (50m)	34.818	40.049	42.064	43.137	43.874	44.469	44.987
10	3.4432	3.9710	4.1891	4.3084	4.3892	4.4520	4.5049
13 (5m)	3.4908	4.0017	4.2121	4.3270	4.4051	4.4659	4.5169
13 (50m)	35.680	40.596	42.481	43.483	44.170	44.720	45.198
20	3.6374	4.1042	4.2830	4.3775	4.4423	4.4940	4.5382
27	3.6836	4.1338	4.3053	4.3965	4.4585	4.5081	4.5507
50	3.7727	4.1911	4.3513	4.4357	4.4932	4.5388	4.5777
100	3.8611	4.2502	4.3987	4.4773	4.5299	4.5716	4.6069
150	3.9089	4.2819	4.4239	4.4995	4.5502	4.5897	4.6234

## Prediction network

The software package used in this study for the modeling is Keras^57^ version 2.4.3, which is an Open Source Library for Neural Network written in Python version 3.8.6 and built on top of TensorFlow^58^ version 2.4.1. The importance of this tools is reducing the role of the physicist to choose an appropriate problem, data scaling and manipulation, DNN architecture, and training technique.

**Figure 2:**
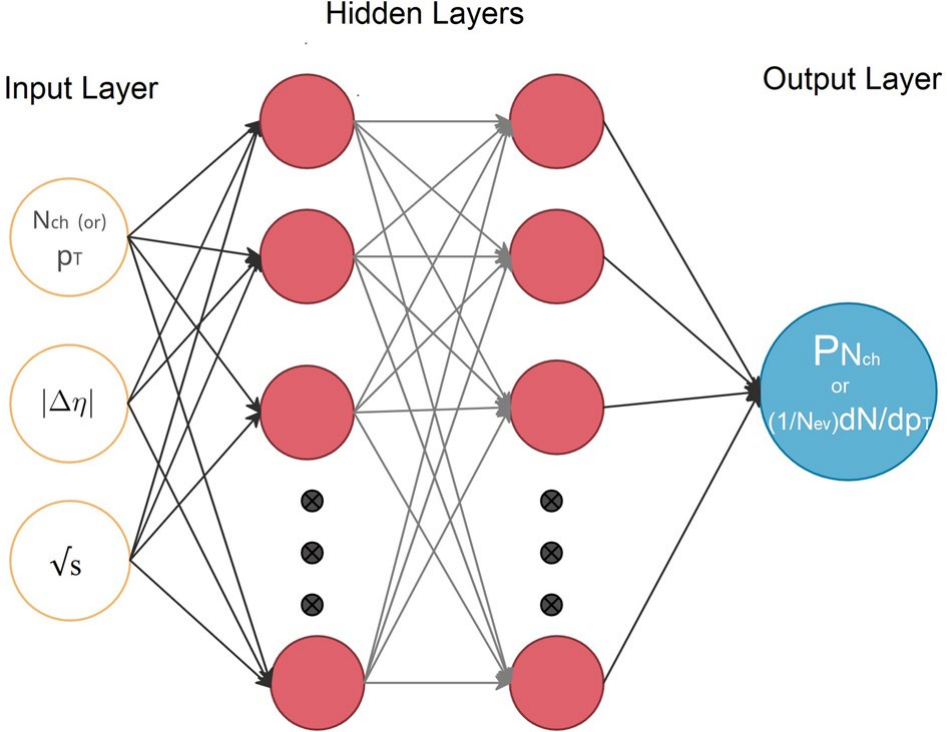
A schematic diagram for our proposed neural network.

Several DNNs were tried to address the problem, with varying number of internal layers and neurons per layer. The DNN model found with inputs $(N_{ch},\sqrt{s},|\Delta \eta |)$ that showed a very good agreement between the probability *P* and the charged-particle multiplicity ($N_{ch}$) at different pseudorapidity windows ($|\Delta \eta |$) and different collision energies ($\sqrt{s}$) consists of an input layer with three inputs, two hidden layers with each 20 neurons and final output layer with only one output, see Fig. 2, and was chosen for this study. This model shows also an excellent agreement for the transverse-momentum ($p_{T}$) distributions but with input ($p_{T}$, $\sqrt{s}$, $|\Delta \eta |$) and the output of the model trained on $(1/N_{ev})dN/dp_T$ which is the distribution giving the number of particles as function of $p_{T}$, divided by the number of events which have at least one particle with $p_{T}>$ 400 MeV within the studied rapidity range.

The initial random weights and biases of the Keras layers are set using the “kernel_initializer” and “bias_initializer” to follow a normal distribution. The activation function implemented for the hidden layers is a hyperbolic tangent “tanh”^59,60^, namely $f(x)= \frac{\sinh (x)}{\cosh (x)}=\frac{e^{x}-e^{-x}}{e^{x}+e^{-x}}$, a nonlinear function to allow for a flexible modeling and the output ranges from $-1$ to 1. Furthermore, the activation function for the output layer is “linear”^60^, namely $f(x)=x$. The role of the activation function is to analyze the total information received by the neuron and this determines the output information produced by the neuron in response to the input information.

The loss value, which quantifies the amount of information lost, used in this model is the mean absolute error (*mae*) between the true value and the predicted one. Mathematically, if $\gamma$ is a vector of *n* predictions, and *Y* is the vector of *n* observed values, then:

3 $$\begin{aligned} mae= \frac{1}{n}\sum _{i=1}^{n}|\gamma - Y| \end{aligned}$$

The optimizer used for this model is the “Adam”^61^ optimizer with a 0.0005 learning rate. This optimizer is used for improving the speed and performance of the training of our model.

We further set the model “batch_size”=100 and in order to avoid over-training, we have used the EarlyStopping class^62^ with min_delta=$e^{-5}$ and “patience” = 1000 in order to stop the processing after the model has reached the smallest loss value for the validation data.

The *pp* collisions generated by PYTHIA at 0.9, 2.36, 2.76, 5, 7 , 8 and 13 TeV are separated into two parts. Two third of the data is used for model training, and the other one-third is used for model validation. The number of events at those energies and different pseudorapidity windows are presented in Table 1 (for the transverse-momentum only 5m data sets are used while for the multiplicity studies the 50m data sets were included).

The best prediction results are obtained when training the multiplicity model with 67% of 0.9, 2.36, 2.76, 5, 7 , 8 and 13 TeV data but in case of the transverse-momentum a better training was achieved, with less bias, using training samples based on the same statistics and hence the samples with 5m collisions each at the different energies were used for this study.

The input values that are used to train the multiplicity model used are $N_{ch} *0.1$, $\sqrt{s}$ and $|\Delta \eta |$ and the output is $P(N_{ch},\sqrt{s},|\Delta \eta |)$. Empirically we found that using a reduced value range for $N_{ch}$ leads to more stable and lower bias results, as it keeps the range of inputs closer to each other, so there is no input intrinsically influencing the model behaviour strongly just as a result of its large value. The multiplicity and $p_T$ distributions cover several orders of magnitude in the bin population, hence for a more stable training procedure and in order to avoid large biases, the training is performed using the logarithms if the bin values for both studies. Furthermore, the number of events with a specific multiplicity must be larger than 10 in order to remove any fluctuations in the spectrum tails for the multiplicity model and the number of particles with a certain $p_T$ is larger than 100 for the transverse-momentum model.

The TensorFlow random seed values are set to one at the start, then the training is deployed until it reaches the value of the smallest loss value compared to validation data, and next the weights and biases that give the least loss are taken. For the comparisons, the results are shown using the original un-scaled values and will be discussed in the next section.

Next, the model is used to predict the energies at future collider energies, e.g. for an upgraded LHC to run at higher energy, i.e. 20 TeV and 27 TeV. Furthermore, this model can be tested for predictions for much higher energies, as expected at the Future Circular Collider (FCC) i.e. 100 TeV. We also test the predictive power for the highest imaginable energy to date for a 100 km ring if the technology would allow for producing 24T instead of 16T magnets superconducting magnets, which would lead to collisions at 150 TeV. Such ideas have been mentioned as a possible –but yet to be demonstrated—upgrade option beyond the baseline for the SPPC machine in the Chinese future collider project proposal^63^.

## Results and discussion

**Figure 3:**
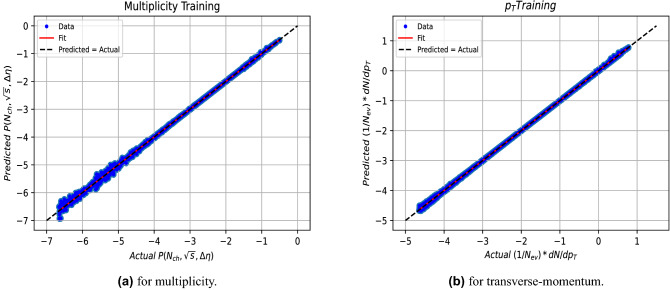
The relation between the predicted and actual output for the training data.

**Figure 4:**
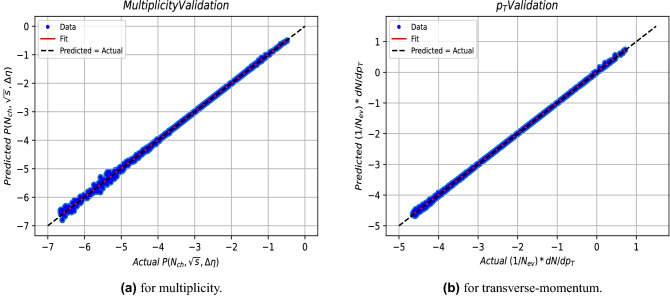
The relation between the predicted and actual output for the validation data.

The performance of the model is found to be excellent for the multiplicity and transverse-momentum distributions, as demonstrated by the relation between the true output from PYTHIA and the one predicted by the model for training data in Fig. 3 and in Fig. 4 for validation data, both shown on a logarithmic scale.

**Table 2:** The fitting parameters regarding the multiplicity and transverse-momentum models both for training and validation represented in Figs. 3 and 4.

	Multiplicity			Transverse-momentum		
	a	b	$R^2$	a	b	$R^2$
Training	0.9992 $\pm$ 0.0004	−0.0020 $\pm$ 0.0014	0.9995	0.9982 $\pm$ 0.0005	−0.0056 $\pm$ 0.0018	0.9994
Validation	0.9981 $\pm$ 0.0005	−0.0037 $\pm$ 0.0021	0.9995	0.9992$\pm$ 0.0006	−0.0028 $\pm$ 0.0024	0.9990

Fits to a linear dependence are made using the fitting equation y=ax+b, where y and x are the predicted and actual values respectively. The fitting parameters (a,b) and $R^2$ are given in the Table 2, where $R^{2}$ is the so called coefficient of determination^64^, which is a measure of the quality of fitting, and defined by:

4 $$\begin{aligned} R^{2}=\frac{\sum \left( \hat{y_{i}}-\bar{y}\right) ^2}{\sum (y_{i}-\bar{y})^2} \end{aligned}$$

where, $y_i$ is the true value, $\hat{y_{i}}$ is the predicted value by the model and $\bar{y}$ is the mean value of all $y_i$ values.

**Figure 5:**
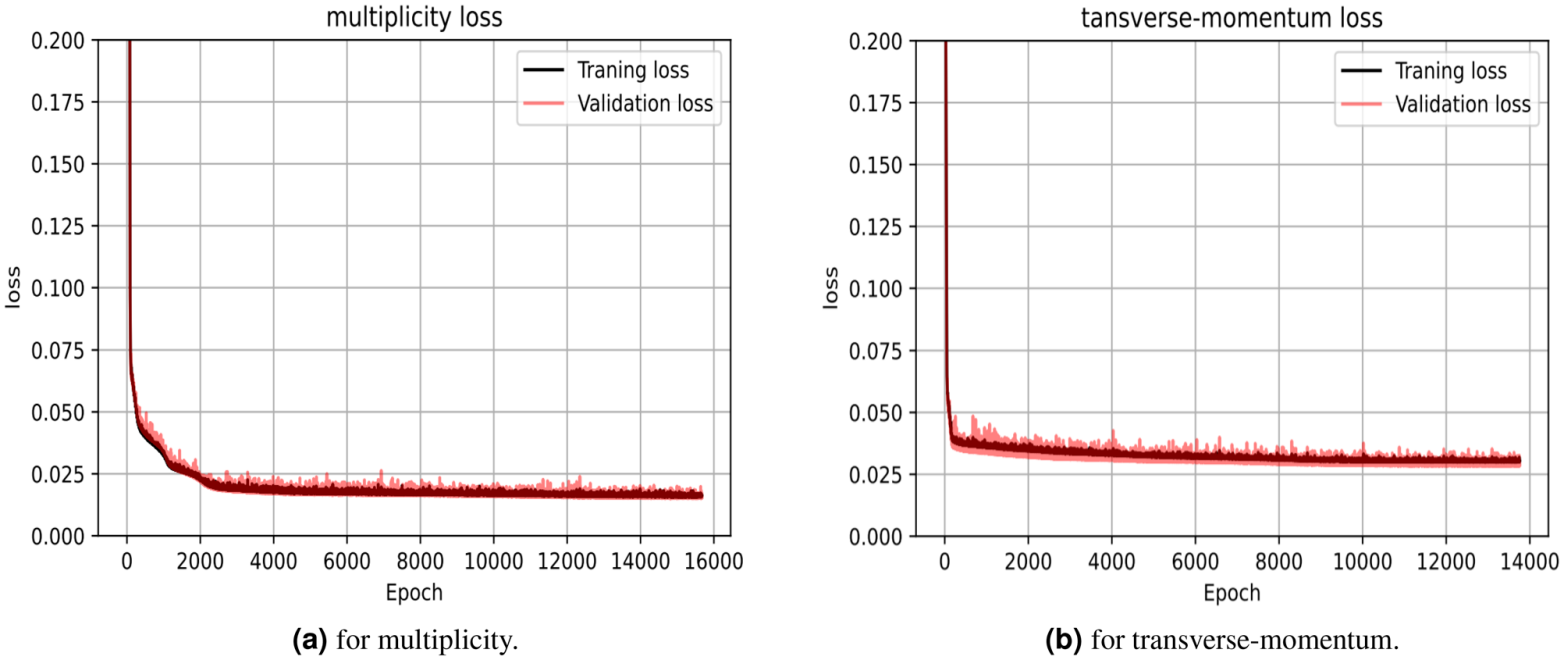
The model training and validation data loss value.

Another important and recommended test of the model quality is shown in Fig. 5 as the loss value of the training and the validation data is almost the same which demonstrates that this model doesn’t suffer from under/over fitting.

**Figure 6:**
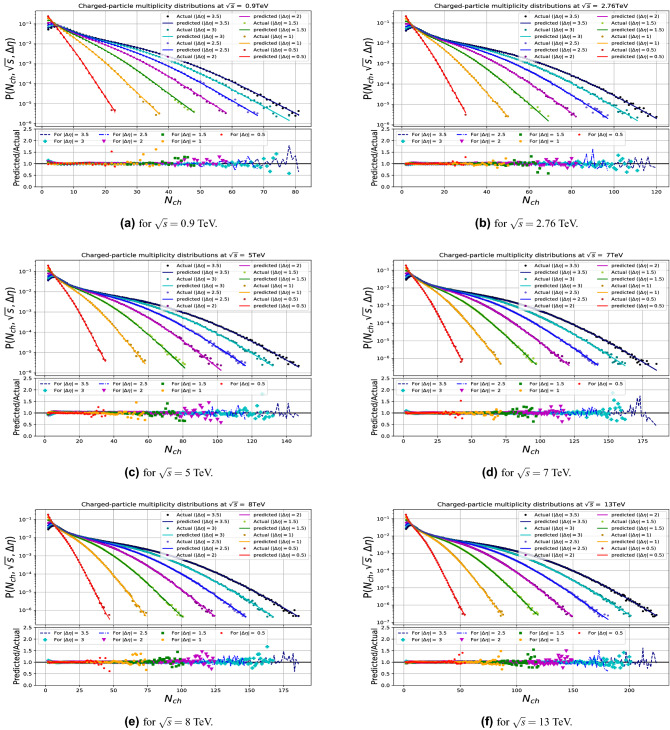
The DNN results in comparison with multiplicity distribution generated by PYTHIA at the training runs (0.9, 2.76, 5, 7, 8 and 13 TeV).

Figures 6 and 8 show the comparisons of the input data with the model predictions for the CM energies used in the training, and demonstrate the quality of the model learning for the multiplicity and transverse-momentum distributions respectively. For the multiplicity distributions the model correctly describes the distributions for all CM energies and pseudorapidity intervals. Expected fluctuations are seen at the high end of the multiplicity distributions due to limited event statistics in the samples. Similarly the transverse-momentum distributions are described with excellent quality, in all demonstrating that the DNN model used has the required flexibility.

The interesting part is now to check how accurate we can “predict” distributions for different CM energies, i.e. which are not included in the training sets. This is checked for a CM energy value within the range of the training sets (10 TeV), and for energy values outside but close to the training range, and values far away from the present range of operation of the LHC. As mentioned before this would be of interest for predictions for either possible new intermediate energy runs of the LHC, for runs with a possible CM energy for an upgraded LHC, or for new future high energy colliders. We do have to assume here that no new as yet unknown physics would set-on at these higher energies, which will significantly impact on these general inclusive variables.

**Figure 7:**
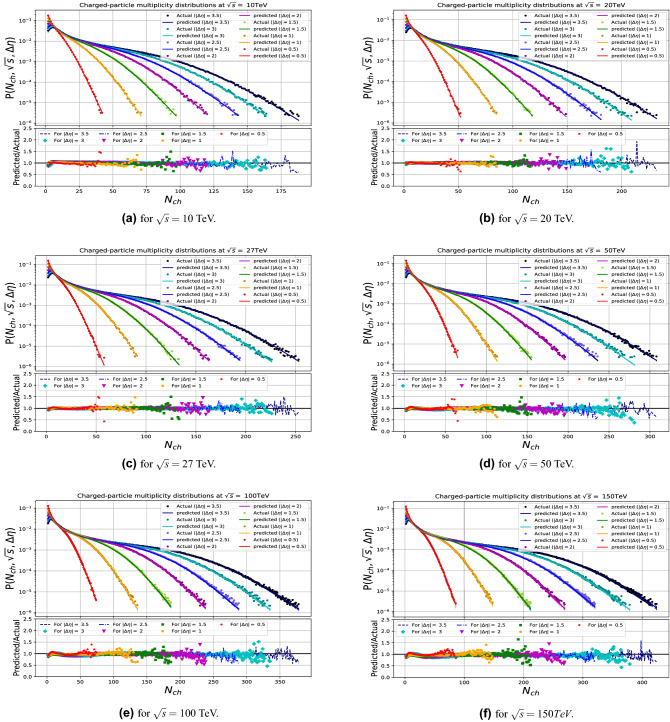
The DNN results in comparison with multiplicity distribution generated by PYTHIA for the untrained runs.

The results are shown in Figs. 7 and 9 and demonstrates that the model gives in general an excellent agreement comparing predicted with the true PYTHIA distributions for CM energies up to 50 TeV, while some modest deviations are seen in case of highest energies tried at 100 and 150 TeV. For the multiplicity predictions in particular, the large $N_{ch}$ end the of distributions are less stable in that region. Similar effects are seen at the high $p_T$ end of the transverse momenta distributions.

In order to test the stability of our model, we have made for the multiplicity studies 50 independent tries, using a different splitting of the data into trained and validated sample and took the average of the tries as well as the envelope of the spread if the results, which are the curves shown on these figures. The small size of the envelope shows that the results are quite stable.

Furthermore, as mentioned before, we have tried a lot of different network configurations, by changing e.g. the number of layers and number of neurons per layer, different activation functions such as (sigmoid, tanh) and different type of optimizers but it appears that the structure that we used in the paper shows the best predictive power.

To check the quality of the predictions we compared the normalized sum of the difference between predicted and observed values for the multiplicity plots. The 10 TeV prediction gives comparable values as the ones from CM energy values used in the training, while the predictions for 50, 100 and 150 TeV are typically a factor of up to maximally 2 worse, but still of acceptable good quality.

A further test of the stability was made on using only two sets of energies 7 (50m) and 13 (50m) TeV and three sets (2.76, 7 (50m) and 13 (50m) TeV) as training sets for composing the multiplicity model. We found the results are already very stable and acceptable for higher energy predictions when using at least three sets of separate and spread-out energy values, see Fig. 10.

**Figure 8:**
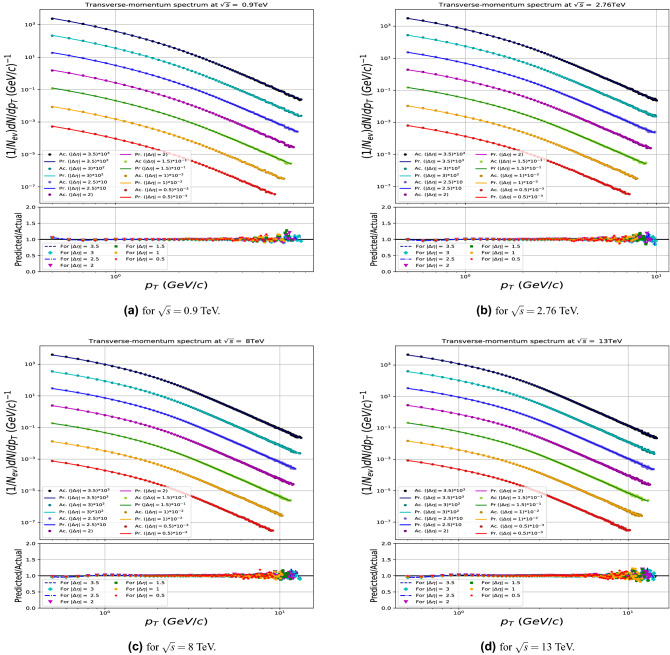
Transverse-momentum spectrum in between the Actual (Ac.) distributions generated by PYTHIA and Predicted (Pr.) by the model in case of the trained data (0.9, 2.76, 8 and 13 TeV).

**Figure 9:**
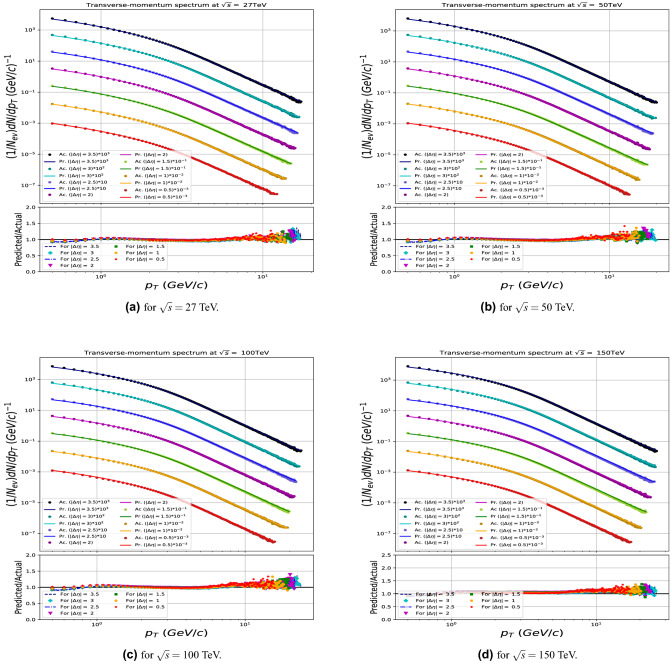
Transverse-momentum spectrum in between the Actual (Ac.) distributions generated by PYTHIA and Predicted (Pr.) by the model in case of the untrained runs (27, 50, 100 and 150TeV).

The final test of the model, to check if it has a bias to the PYTHIA generator, has been done using EPOS-LHC and HERWIG. The same technique has been applied with $p_T>$ 100 MeV/c. Because EPOS and HERWIG has larger generation time we have generated about 900k events at every energy just to check the model prediction at different generators. The model showed a relative good prediction with respect to the smaller number of training data both for EPOS and HERWIG, see Fig. 11.

The network structure of our model is of the form [3x20x20x1] for the structure in the different layers. We note that the output of this model can in principle directly be obtained by multiplying the data matrices with the derived weighting matrices and adding biases for each layer, which can be represented by the following equation:

5 $$\begin{aligned} Y^{[1x1]}=f_3(f_2(f_1(X^{[3x1]}*W_1^{[20x3]}+B_1^{[1x20]})* W_2^{[20x20]}+B_2^{[1x20]})*W_3^{[1x20]}+B_3^{[1x1]}) \end{aligned}$$

where $Y^{[1x1]}$ is the output of our presented model, i.e. $P(N_{ch},\sqrt{s},|\Delta \eta |)$ in case of multiplicity and $(1/N_{ev}).dN/dp_T$ in case of $p_{T}$ modeling; $X^{[3x1]}$ is the input matrix, i.e. $N_{ch} *0.1$, $|\Delta \eta |$ and $\sqrt{s}$ for multiplicity and $p_{T}$, $|\Delta \eta |$ and $\sqrt{s}$ in case of transverse-momentum. Here $f_1$,$f_2$ are the activation functions of the hidden layers which are the hyperbolic tangent functions (tanh) and $f_3$ is the activation function of the output layer, a first-order polynomial. The matrix $W_1^{[20x3]}$ is a 20 by 3 matrix representing the weights for the first hidden layer neurons, $W_2^{[20x20]}$ is 20 by 20 matrix for the second hidden layer neurons and $W_3^{[20x1]}$ for the output layer. $B_1^{[1x20]}$ and $B_2^{[1x20]}$ are 1 by 20 matrices representing the biases for the first and second hidden layers and $B_3^{[1x1]}$ is for the output layer neuron. These matrices can be found in ^65^.

**Figure 10:**
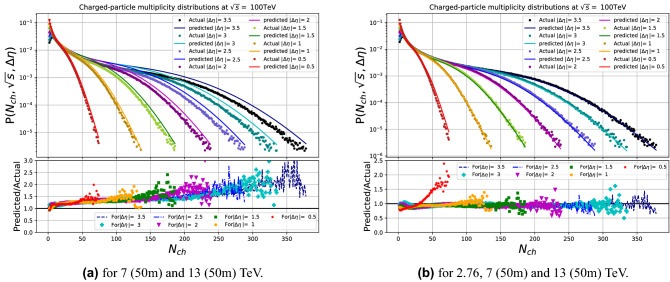
Test of the multiplicity model prediction at 100 TeV when training on different number of energies.

**Figure 11:**
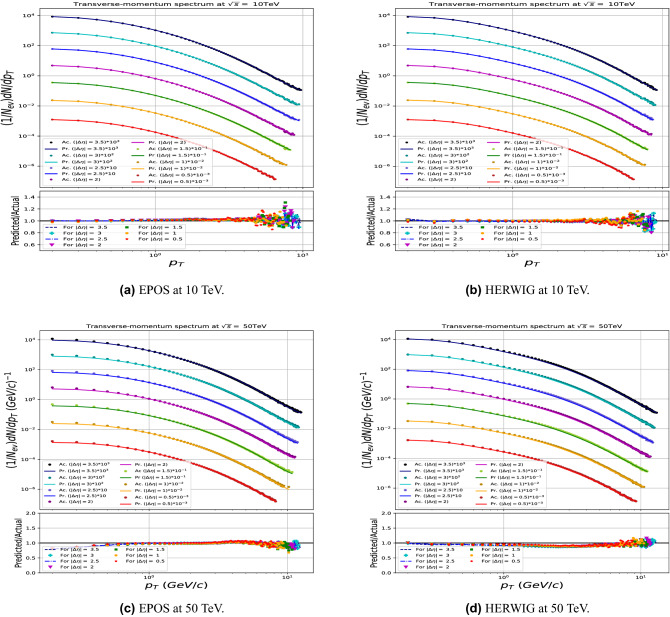
The $p_T$ prediction using the same model trained on different event generators.

## Conclusion

We deploy machine learning techniques to build a model for the description of charged-particle multiplicity and transverse-momentum measurements in high energy *pp* interactions. Proton-proton collisions have been generated by the event generator PYTHIA at the energies at which the LHC operated so far to train the model and test its predictive power. A good ML structure that shows small loss value and leads to highly stable predictions has been reported.

The model with the [3-20-20-1] structure, and tanh activation function in the hidden layer and a linear function for the output layer, shows an excellent agreement in comparison with the trained and untrained runs for all the seven pseudorapidity windows selected, with the coefficient of determination (see eqn. (4)) up to 0.9995 in case of multiplicity and about 0.9990 in case of $p_{T}$.

This model succeeded in providing good predictions for the charged-particle multiplicity and transverse-momentum distributions at new center of mass energies. Hence such a procedure, when applied on real measured data at the LHC at the different energies could be used in studies for possible future CM energies, at the LHC or future hadron colliders, to give an initial idea of the to be expected particle density in future experiments.Also, the model was tested by using Herwig and EPOS-LHC, it succeeded to get good prediction with respect to small number of generated events.
